# Ethanol Sclerotherapy for Endometriomas in Infertile Women: A Narrative Review

**DOI:** 10.3390/jcm13247548

**Published:** 2024-12-11

**Authors:** Yavuz Emre Şükür, Batuhan Aslan, Bulut Varlı, Pınar Özcan, Angelos Daniilidis, Dimitrios Rafail Kalaitzopoulos

**Affiliations:** 1 Department of Obstetrics and Gynecology, Reproductive Health Center, School of Medicine, Ankara University, 06620 Ankara, Türkiye; yesukur@gmail.com (Y.E.Ş.); drbatuhanaslan@gmail.com (B.A.); bulutvarli@gmail.com (B.V.); 2 Graduate School of Health Science, Ankara University, 06110 Ankara, Türkiye; 3 Department of Obstetrics and Gynecology, Ankara Health Science University, Etlik Zubeyde Hanim Women’s Health and Research Hospital, 06010 Ankara, Türkiye; 4 Department of Obstetrics and Gynecology, School of Medicine, Bezmialem University, 34093 İstanbul, Türkiye; drpinarozccan@hotmail.com; 5 1st Department of Obstetrics and Gynecology, Papageorgiou General Hospital, Faculty of Medicine, School of Health Sciences, Aristotle University of Thessaloniki, 541 24 Thessaloniki, Greece; angedan@hotmail.com; 6 Department of Obstetrics and Gynecology, District Hospital of Schaffhausen, 8208 Schaffhausen, Switzerland

**Keywords:** cystectomy, endometrioma, ethanol sclerotherapy, in vitro fertilization, ovarian reserve, pregnancy

## Abstract

Ethanol sclerotherapy (EST) has gained attention as a minimally invasive treatment option for ovarian endometriomas, particularly in infertile women with endometrioma undergoing in vitro fertilization (IVF). Endometriomas are associated with decreased ovarian reserve and impaired fertility outcomes, and traditional surgical approaches, such as cystectomy, often lead to further reductions in ovarian reserve. Ethanol sclerotherapy offers a potential alternative that preserves ovarian function while effectively managing endometriomas. This review examines the safety, efficacy, and impact of EST on ovarian reserve, IVF outcomes, and recurrence rates. Comparative studies suggest that pregnancy rates following EST are similar to or better than those after cystectomy, with the added benefit of more oocytes retrieved, which may lead to higher cumulative live birth rates. Despite these promising results, challenges such as recurrence and complications, particularly with prolonged ethanol exposure, remain. The use of transvaginal versus laparoscopic approaches and optimal ethanol exposure protocols are areas of ongoing research. The need for further large-scale, prospective studies is highlighted to refine the EST protocol and better understand the long-term outcomes. Sclerotherapy presents a feasible option for preserving fertility in women with endometriomas, with positive implications for IVF success and ovarian reserve preservation.

## 1. Introduction

Endometriosis is an estrogen-dependent, chronic, and inflammatory disease characterized by the ectopic presence of endometrial stroma and glands in extrauterine tissues [1]. Since there is still no reliable and non-invasive gold standard method for the diagnosis of endometriosis, its exact prevalence cannot be known, but it is estimated to be up to 10% in the general female population and up to 50% in infertile women [2,3]. The prominent symptom is pain in about half of the patients, while it is infertility in the other half [4]. Although the relationship between endometriosis and infertility is clinically clear, the etiopathogenesis of infertility in endometriosis patients is a complex, multifactorial, and controversial area [5]. There are multiple mechanisms that cause infertility including anatomical changes in the pelvic cavity, uterus, and ovaries, which may cause impaired oocyte, sperm, and embryo transport, impaired folliculogenesis, granulosa cell dysfunction, impaired immune functions in follicular and peritoneal fluid, impaired sperm function, and fertilization and implantation defects. The effect of endometriosis on oocyte quality is controversial [6]. Although data are available only from assisted reproductive technology (ART) studies, endometriosis has been shown to be associated with reduced oocyte quality due to regulatory mechanisms involved in steroid metabolism and biosynthesis, oxidative stress response, and cell cycle [7]. Endometriosis may also cause differentiation in the eutopic endometrial tissue [8]. The receptivity of endometrium in endometriosis is also a controversial issue. However, abnormal inflammation, in situ estrogen production, and progesterone resistance are the main factors related to decreased receptivity [9].

Endometriomas may cause a reduction in the available functional ovarian tissue by the space-occupying effect and/or local reaction [10]. An endometrioma itself may cause premature follicle recruitment, higher rates of atresia, and a lower quality of the remaining primordial follicular pool [11]. According to the burn-out hypothesis, this local inflammatory status may cause the dysregulation of folliculogenesis and an enhanced recruitment and atresia of primordial follicles, which can occur even in small cyst sizes [11]. Hence, the removal of endometrioma cysts may improve ovarian cortex milieu and can potentially have a positive impact on ovarian function. In addition, during endometrioma surgery, the removal or destruction of endometriotic foci that could not be detected by preoperative imaging and the removal of possible adhesions may also have a positive impact on the chance of spontaneous conception. However, endometrioma surgery has a significantly negative impact on ovarian reserve. The most recent systematic review and meta-analysis showed a significant serum anti-Müllerian hormone (AMH) level drop in the early, intermediate, and late terms following both unilateral and bilateral endometrioma surgeries [12]. According to this analysis, 9–12 months after surgery, serum AMH levels decrease by 39.5% in unilateral cystectomy and 57% in bilateral cystectomy [12]. These values seem to be far above the expected natural decline in serum AMH levels in a non-selected population [13]. On the other hand, endometrioma itself may cause a decline in serum AMH levels. In a prospective longitudinal study, 40 women with endometrioma and 40 age-matched healthy controls were assessed for AMH levels and antral follicle counts at the baseline and 6 months after recruitment [14]. The median decline in AMH levels were 26.4% and 7.4% in the endometrioma and control groups, respectively, and the decline was more prominent in cases of bilateral endometrioma [14].

To summarize briefly, endometrioma in situ during an IVF cycle may cause lower numbers of oocytes to be collected, which may in turn result in lower number of embryos to be transferred and lower cumulative live birth rates [15]. On the other hand, a recent meta-analysis failed to demonstrate a negative effect on oocyte and embryo qualities [15]. However, another recent meta-analysis of donor oocyte cycles found a significant negative impact of endometriomas on endometrial receptivity and implantation rates [16]. Laparoscopic cystectomy has the potential to improve a possible negative impact on quality and implantation. However, the surgical resection of endometriomas significantly decreases ovarian reserve and may further diminish the cumulative live birth rate [12]. In these circumstances, ethanol sclerotherapy (EST) has the potential to eliminate endometriomas while also preserving ovarian reserve.

## 2. History of Ethanol Sclerotherapy for an Endometrioma

Ethanol sclerotherapy for an endometrioma was first described by Akamatsu et al. in 1988, with six procedures performed on five patients [17]. The initial approach involved aspiration followed by 30 min of alcohol exposure [17]. Post-aspiration tetracycline, methotrexate, and lauramacrogol applications were also described in several publications [18,19,20,21,22,23]. However, ethanol is the most common agent used. Sclerotherapy was suggested as a promising approach in 1997 as part of an ART program for recurrent endometriomas after medical and surgical treatment [24]. Subsequently, laparoscopy following alcohol injection revealed filmy adhesions involving the surrounding organs in four patients. It remains unclear whether these adhesions resulted from sclerotherapy or were previously present [25]. Although the first publication dates back four decades, its use has increased, and related studies have accelerated in the last 5 years. Ethanol sclerotherapy continues to be a popular option, particularly due to its potential protective effect on ovarian reserve compared to cystectomy. However, practitioners harbor concerns regarding recurrence, infection, and uncertainty about the mechanism of action. This review aimed to examine the potential role and effectiveness of EST in the IVF practices of infertile patients with endometrioma.

## 3. Technique and Mechanism of Ethanol Sclerotherapy

Transvaginal EST can be performed with the patient in the lithotomy position under either local anesthesia/sedation or general anesthesia. The procedure begins with the application of vaginal iodine and the administration of antibiotic prophylaxis. Under transvaginal ultrasound guidance, the endometrioma is punctured, and its contents are aspirated. The cyst is then flushed with saline until the returning fluid runs clear. Following this, 96.5% ethanol, amounting to 60% of the aspirated cyst volume, is injected into the cyst. The ethanol is retained for 10 min and then aspirated to complete the procedure.

Sclerotherapy is a process that utilizes agents to break down target tissue. The resulting effect of the agent may lead to the formation of hardened tissue [26]. Sclerotherapy can permanently disrupt the endothelium of target structures, thereby limiting recurrence, proliferation, or collateralization [26]. Ethanol is the primary agent used in sclerotherapy and is also the primary agent used for the comparative evaluation of other sclerosing agents. The effect of ethanol, which is commonly used in radiological approaches, on the epithelium is well-documented. Its mechanism of action involves a combination of cytotoxic damage induced by denaturation and the extraction of surface proteins, the hypertonic dehydration of cells, and coagulation and thrombosis when blood products are present [26]. It was hypothesized that EST creates a controlled inflammation, stimulates fibroblast proliferation, and causes cellular dehydration, leading to fibrinoid necrosis and subsequent cyst wall solidification, hardening, adhesion, and closure [26].

Endometriosis is known to be an inflammatory condition, and reducing inflammation might be beneficial. Aliabad et al. specifically investigated the impact of EST on pro-inflammatory cytokine levels. While the study observed differences in certain cytokine levels (IL-1β and IL-6) between women with and without endometriosis, they did not find a significant change in the inflammatory marker levels after sclerotherapy [27]. However, this may be due to the localized effects of EST.

The effectiveness of EST depends on variables such as the percentage of ethanol used and the duration of exposure. Traditionally, 96% ethanol is preferred for endometrioma sclerotherapy. However, the specific volume applied and the duration of exposure varies. Additionally, both transvaginal and laparoscopic approaches have been used for ethanol application, though their comparative superiority has not been established yet. Transvaginal EST entails aspiration of the endometrioma via the vaginal route using a single lumen needle, followed by alcohol injection [28,29]. In contrast, the laparoscopic approach involves direct trocar-assisted puncture and aspiration of the endometrioma, again followed by alcohol injection. While the underlying mechanism of action is essentially the same, the laparoscopic approach offers the advantage of allowing for the evaluation of the pelvis and potential surgical intervention for other pelvic pathologies. Conversely, the vaginal approach is generally considered a less costly and less invasive alternative.

## 4. Ethanol Sclerotherapy in the IVF Treatment of Infertile Women with Endometrioma

Ethanol sclerotherapy is a widely used method in patients with endometrioma. While the effects of this approach on pain scores, ovarian reserve, endometrioma size, and IVF outcomes have been extensively studied, the lack of randomized controlled trials is striking. Herein, EST prior to IVF was compared with cystectomy prior to IVF or with IVF in the case of endometrioma in situ.

### 4.1. EST vs. Cystectomy Prior to IVF

Several previous studies have compared EST with cystectomy prior to IVF treatment. In 2009, Yazbeck et al. investigated the use of EST in a specific context: treating recurrent endometriomas in infertile women before ovarian stimulation. Their findings indicated that EST resulted in improved ovarian reserve and response to stimulation compared to a control group who underwent laparoscopic cystectomy. Furthermore, the EST group demonstrated higher clinical and cumulative pregnancy rates [30]. In a retrospective study, Lee et al. directly compared the in vitro fertilization (IVF) treatment outcomes between three groups: surgical resection, EST, and endometrioma in situ. They found that women who underwent surgical resection of endometriomas had a significantly lower total AFC compared to the aspiration and control groups. Despite the difference in AFC, there were no statistically significant differences in most IVF outcomes between the three groups. This includes the number of cycles initiated, number of oocytes retrieved, and fertilization rates [31]. However, these studies were limited by small sample sizes and technical variations. Alborzi et al. conducted a prospective study comparing EST and laparoscopic cystectomy in 101 infertile women. While they found no significant differences in IVF outcomes like pregnancy and live birth rates between the two groups, their study revealed a significantly higher recurrence rate of endometriomas in the sclerotherapy group. This highlights a crucial consideration for patients and clinicians about recurrence [32]. However, this study used a technique where ethanol was left in the cyst and not aspirated, raising questions about the potential negative impact of prolonged alcohol exposure. Conversely, Antonaci et al. compared cystectomy and EST before IVF and reported significantly higher cumulative pregnancy and live birth rates as well as better ovarian reserve parameters in the EST group after six months of follow-up [33]. In addition, no difference in MII oocyte and embryo quality was observed, while a lower pregnancy loss rate was observed in the EST group. In this study, the patient impression of global improvement (PGI-I) questionnaire revealed that the success rates were 91% in the EST group and 69.3% in the laparoscopic cystectomy group.

In short, there is no randomized controlled trial comparing EST with cystectomy before IVF. All observational studies are limited due to small sample sizes and reported outcome parameters. Since both methods eliminate the existing endometrioma, similar effects per embryo transfer are expected, and all but one reported similar clinical pregnancy or live birth rates for the two methods. However, when the cumulative live birth outcomes are evaluated, more successful results were achieved with EST, which preserves the ovarian reserve, leading to the retrieval of more oocytes. Studies comparing EST with cystectomy are summarized in Table 1.

### 4.2. EST Prior to IVF vs. IVF with In Situ Endometrioma

The potential advantages of EST prior to IVF, compared to directly performing IVF, have been examined in several studies. Suganuma et al. categorized infertile women with ovarian endometrial cysts undergoing IVF-ET into three groups based on the type of pretreatment: cystectomy, cyst aspiration, and no treatment. In the cyst aspiration group, the procedure was followed by treatment with or without alcohol fixation. Their findings indicated no significant difference in the quality of the oocytes collected among the groups; however, the fertilization rates improved following cyst aspiration [37]. Although EST was not directly evaluated, the observed improvement in fertilization rates following cyst aspiration suggests that this step may play a critical role in improving IVF outcomes. Petroza et al. assessed the aspiration of endometriomas with ethanol sclerosis and saline and focused on the impact on follicle and oocyte numbers [38]. According to their results, EST led to a higher peak estradiol level and a greater increase in the number of oocytes retrieved from the initial to post-aspiration IVF cycle compared to saline. The only RCT assessed the efficacy of aspiration and ethanol injection for recurrent endometrioma before an IVF cycle [34]. The authors found that EST was effective in reducing the endometrioma recurrence rates and observed significantly higher oocyte retrieval rates, but no significant difference was observed in the pregnancy outcomes between groups [34]. While the findings demonstrate the potential benefits of EST, the authors acknowledged the limitations of the study’s small sample size and called for further research. In addition, a recent retrospective study reported that EST before IVF was associated with a 2.68-fold increase in cumulative LBR in women with moderate-severe endometriosis (95% CI 1.13–6.36, *p* = 0.02) [35]. Beyond the primary outcome, EST also significantly improved the clinical and biochemical pregnancy rates. Finally, Rabattu et al. found that the pregnancy and live birth rates were significantly higher in patients who underwent EST prior to IVF compared to those managed directly with IVF (weighted OR 2.9, 95% CI 1.4–6.6 and weighted OR 2.4, 95% CI 1.1–5.4; respectively) [36]. The authors also reported a significantly lower rate of pregnancy loss (weighted OR 0.3, 95% CI 0.1–0.9). The studies that compared IVF outcome between women who underwent EST prior to IVF and those who underwent IVF directly with in situ endometrioma are summarized in Table 1.

In 2017, Cohen et al. conducted a systematic review and meta-analysis and found that the number of oocytes retrieved was higher after sclerotherapy compared to laparoscopic cystectomy, but there was no significant difference in the clinical pregnancy rates between the two procedures [39]. Additionally, there was no difference in the oocyte retrieval or pregnancy rates between women who underwent sclerotherapy and those who did not. A meta-analysis conducted by Kim et al. found that that sclerotherapy, regardless of duration, was associated with a significantly higher pregnancy rate compared to surgery [40]. Recently, a systematic review and meta-analysis by Ronsini et al. revealed that sclerotherapy demonstrated a comparable effectiveness to surgery in resolving endometriomas, with a lower risk of complications [41]. Furthermore, there were no significant differences in the pregnancy rates between the two treatment approaches. These findings strongly support the use of sclerotherapy as a safe and effective alternative to surgery in the management of endometriomas [41]. However, considering the heterogeneity of the included studies and technical differences, the level of evidence for these comparisons should be questioned. The differences in the methodologies used, the small sample sizes, and the absence of randomized controlled trials are particularly noteworthy [41].

To date, very few studies and meta-analyses have compared the use of EST before IVF with direct IVF application. All of these studies have found either a positive effect of EST or no negative impact. However, there are some important points to consider when interpreting the results of these studies. First, the studies are retrospective in nature and have small sample sizes. It is noteworthy that only one RCT has been published up to date [34]. Second, non-standardized and varying EST techniques have been applied. There are differences among studies in parameters such as the size of the intervened endometrioma, the volume of ethanol used, and the duration of ethanol application. Moreover, most studies have presented their results in terms of clinical or live birth rates. However, in such cases, presenting cumulative outcomes is crucial to reveal the true positive effect of EST. The most expected benefit of sclerotherapy is the increase in the number of oocytes and embryos, and the most accurate way to evaluate these increases is through cumulative rates.

## 5. Ethanol Sclerotherapy and Natural Conception

Although many previous studies have reported pregnancy rates, most have not differentiated between natural and IVF pregnancies. Only a few studies have specified spontaneous pregnancy rates following EST (Table 2) [42,43,44,45,46]. However, the main issue with these studies is that their primary objective was not to assess the chance of spontaneous pregnancy, and the spontaneous pregnancy rate was considered as a secondary outcome. Furthermore, all studies were retrospective in nature. In these studies, the issue of lack of standardization from a technical perspective was observed. All but one of the studies employed transvaginal ultrasound-guided sclerotherapy [45].

However, aside from the older studies, more recent ones have reported endometrioma sizes and all have performed procedures on endometriomas larger than 3.5 cm. Only a small number of patients with a desire for fertility or a history of infertility could follow the procedure, and specific pregnancy rates for these patients were provided. According to the results of these five studies, the spontaneous pregnancy rates in women with known infertility or a desire for fertility ranged from 42.8% to 52.1%, which is comparable to the spontaneous pregnancy rates after laparoscopic cystectomy [42,43,44,45,46,47,48,49]. In summary, although there is a low level of evidence, spontaneous pregnancy rates at least equivalent to surgery can be achieved after ethanol sclerotherapy.

## 6. Ethanol Sclerotherapy and the Safety Issues

### 6.1. Recurrence Rates

The majority of studies have examined the recurrence and complication rates of EST including its impact on ovarian reserve. Recurrence rates of EST are typically compared with cystectomy and vary widely, ranging from 0 to 50%, as reported within a follow-up period of 6–36 months (Table 3). However, in general, one year recurrence rates are reported to be below 20%. In almost all of the comparative studies, similar recurrence rates were observed between EST and laparoscopic cystectomy [33,43,50,51,52]. Only Alborzi et al. reported higher recurrence rates with the EST procedure when compared to cystectomy, which may be due to technical differences [32]. In the most recent comparative study, EST was performed transvaginally with ethanol left in place for 10 min [53]. According to the results of this study, the recurrence rate was 11.4% in the EST group compared to 15.9% in the cystectomy group [53].

Variations in the EST technique, follow-up periods, and additional post-procedure treatments across studies make it difficult to interpret their results collectively. The study by Noma and Yoshida highlighted the importance of the duration of ethanol exposure, reporting a significantly lower recurrence rate when ethanol was left in place for 10 min or longer [43]. Interestingly, a higher recurrence rate was observed in women with multiple endometriomas compared to those with a single cyst, suggesting that multiple endometriomas may be a potential risk factor for recurrence. Similarly, in a recent meta-analysis including 28 studies and 1301 patients, the duration of ethanol exposure was assessed [40]. Direct comparisons showed that the recurrence rate was significantly lower with sclerotherapy >10 min than with sclerotherapy ≤10 min (OR, 0.2; *p* = 0.015) [40].

### 6.2. Complication Rates

The EST procedure for endometriomas is a less invasive alternative to cystectomy. However, it carries the risk of severe complications, such as pelvic abscesses, although these complications are poorly documented. A recent prospective cohort pilot study reported fewer complications with EST compared to cystectomy [50]. No serious adverse effects (Clavien–Dindo ≥ III) were observed in the EST group. Additionally, EST was significantly more cost-effective than laparoscopic surgery for treating endometriomas, with a notably shorter median hospital stay.

In a recent retrospective study, Miquel et al. reported and described the failures and complications of transvaginal EST [64]. The authors noted complications in 9.9% of EST procedures, with most being low-grade according to the Clavien–Dindo classification. Two patients (1.6%) experienced grade III complications (ovarian abscess), one patient (0.8%) had a grade IV complication (alcohol intoxication), and no patients experienced grade V complications [64]. The failure rate, defined as incomplete procedures due to leakage or aspiration failure, was 10.3%.

Ethanol sclerotherapy is associated with a high safety profile [65]. Most complications reported were minor such as fever, abdominal pain, and pelvic pain. According to a large meta-analysis, the pooled major complication rate for ultrasound-guided sclerotherapy was only 1.7%, and it was not related to the duration of ethanol exposure [40]. In addition, the minor complication rate was estimated to be 11%, and subgroup analysis showed that sclerotherapy >10 min had a pooled minor complication rate of 14.1%, which was higher than that of 5.4% for sclerotherapy ≤10 min (*p* = 0.048) [40].

### 6.3. Impact on Ovarian Reserve

One of the major concerns regarding EST is the potential damage to ovarian reserve and its subsequent impact on fertility outcomes. Previous studies have primarily assessed the effect of EST on ovarian reserve by evaluating changes in AMH levels, with some also reporting on antral follicle counts (AFCs). Table 3 summarizes the impact of the EST procedure on ovarian reserve including comparisons with cystectomy. Most studies indicate that ethanol sclerotherapy does not significantly affect ovarian reserve.

Koo et al. found that catheter-directed sclerotherapy with 99% ethanol was associated with significantly better preservation of ovarian reserve compared to surgical excision of endometriomas [51]. They demonstrated that serum AMH levels remained stable 6 months after EST. A prospective multicenter pilot cohort study by Martinez-Garcia et al. suggests that EST may have a less detrimental impact on ovarian reserve compared to surgery, as evidenced by a smaller reduction in AMH levels and significant increases in serum estradiol concentrations, with possible AFC recovery [46].

Lee et al. reported minimal detrimental effects on ovarian reserve, even in cases of recurrent endometriomas [66]. Huang et al. specifically investigated the effect of ethanol sclerotherapy on AMH levels [67]. They observed a significantly greater decline in AMH levels at six months follow-up in the ethanol retention group compared to the non-retention group. This finding suggests that while ethanol retention might be more effective in preventing recurrence, it could have a greater negative impact on ovarian reserve.

In a separate study, Lee et al. evaluated the efficacy of EST in patients with low pre-procedural AMH levels and found that EST effectively reduced pain and the serum CA-125 levels without further diminishing the AMH levels [60]. However, it is important to note that this was a preliminary study with a relatively small sample size.

## 7. Discussion

The management of endometriomas remains a subject of debate, particularly due to the negative impact of cystectomy on ovarian reserve, prompting fertility specialists to explore alternative approaches. Ethanol sclerotherapy has gained renewed interest and could be considered for appropriate patients. The comparable recurrence rates between ethanol sclerotherapy and laparoscopic cystectomy suggest that sclerotherapy could offer similar therapeutic effects while potentially minimizing ovarian damage, an important consideration for women aiming to preserve fertility.

Current evidence indicates that EST is a safe and effective treatment option for ovarian endometriomas in IVF patients. When comparing IVF outcomes, pregnancy rates with EST can be similar to or even better than those with surgery. However, according to a limited number of comparative studies, performing EST before IVF is more successful than proceeding directly with IVF while leaving the endometrioma in place. The main advantage of EST in patients undergoing IVF is that it allows for the retrieval of more oocytes, which in turn leads to the creation of more embryos. This can potentially increase the cumulative live birth rate.

The main challenges with ethanol sclerotherapy are recurrence, complications, and potential negative effects on ovarian reserve. Recurrence rates similar to those seen after surgery have been observed, particularly in studies where alcohol exposure lasted 10 min or more, suggesting that standardizing the EST protocol could improve its success. The minimal impact of EST on ovarian reserve is a key advantage over cystectomy. EST has also been associated with low complication rates in the literature. Its short hospital stay and positive effects on symptoms such as dyspareunia and dysmenorrhea may make it a preferable option for many patients. On the other hand, EST may prolong the waiting period for IVF when compared to IVF performed with the endometrioma left in situ. Additionally, favorable outcomes have been reported in patients with recurrent endometriomas post-surgery and those with low ovarian reserve, further enhancing the reliability of EST. However, there are no specifically established inclusion and exclusion criteria for sclerotherapy in the literature. Most studies have performed ethanol sclerotherapy (EST) for endometriomas measuring 3–10 cm in diameter (Table 1, Table 2 and Table 3). The presence of multiple or bilateral endometriomas is not typically considered as an exclusion criterion.

Although EST has become widely practiced and appears to be a feasible method, there are still some unresolved issues. One of these concerns is the exclusion of malignancy. Endometriosis may present malignant transformation occurring under the influence of local inflammatory, vascular and hormonal factors, and by the alteration of tumor suppressor proteins and the inhibition of cell apoptosis, with an increased degree of lesion proliferation [68,69]. Several proinflammatory/vascular/hormonal changes such as increased expressions of cytokeratin, CD34, CD68+ macrophages, increased Ki67, and inactivation of tumor suppressor genes may trigger endometriosis progression and malignant transformation [68]. Especially with transvaginal sclerotherapy, malignancy may not be properly assessed. Even if the aspirated fluid is sent for cytological examination, it may not be sufficient. It should be noted that in laparoscopic sclerotherapy, there is the advantage of direct observation and the possibility of taking a biopsy. While CA125 levels may be elevated in patients with endometriomas, this marker alone has limited diagnostic value in assessing malignancy. In our view, ultrasonographic evaluation of the cyst is crucial, and any suspicion of malignancy should preclude sclerotherapy and necessitate surgical intervention. Although the absolute risk is not high, the risk increases when the endometrioma is >9 cm or in patients older than 45 years old. Additionally, nodularity and enlarging cysts may indicate malignant transformation [69].

Another concern is the potential for high-concentration alcohol to leak and cause adhesions in the peritoneum and tubal mucosa, a situation that has not been clearly evaluated in any study thus far. Therefore, in patients already undergoing IVF treatment, the transvaginal approach may be acceptable. However, for patients who aim to conceive spontaneously, laparoscopic sclerotherapy may be more appropriate, as any alcohol leakage can be immediately aspirated, and the peritoneal cavity can be washed. Another underexplored aspect of sclerotherapy is the type of sclerosing agent used. Limited studies in the literature have reported the use of substances such as methotrexate, tetracycline, and lauromacrogol for sclerotherapy, but sufficient comparative data from these studies are lacking [18,20,21,22,23]. Fisch et al. reported that 75% of patients achieved complete resolution after sclerotherapy with 5% tetracycline, with a 57% ongoing pregnancy rate observed in 28 patients who underwent IVF [18]. However, this study did not include a control group. Similarly, Shawki et al. reported a 42.8% live birth rate following IVF in 65 patients with unilateral endometrioma treated with sclerotherapy using methotrexate, but this study also lacked a control group [22]. A critical limitation in the current literature is the absence of direct comparisons between these agents or with ethanol, leaving a gap in understanding their relative effectiveness and safety.

Nevertheless, the optimal protocol for ethanol sclerotherapy including the ethanol concentration, volume, and duration of exposure is still under investigation. While some studies suggest that longer ethanol retention times are associated with a lower recurrence rates, they may also decrease the ovarian reserve. To better understand the long-term safety and efficacy of ethanol sclerotherapy, larger, prospective studies with extended follow-up periods are needed. Future research should also incorporate patient-reported outcomes such as pain relief, quality of life, spontaneous pregnancy rates, and cumulative IVF outcomes as important endpoints.

## 8. Conclusions and Future Directions

Ethanol sclerotherapy shows promise as a treatment for ovarian endometriomas in IVF patients. It effectively reduces the size of endometriomas and alleviates associated symptoms without significantly compromising the ovarian reserve. The choice between sclerotherapy and surgical excision should be individualized, considering factors such as the patient’s age, fertility goals, symptom severity, and the size and characteristics of the endometrioma. Notably, the results of an upcoming multicenter randomized controlled trial evaluating the efficacy of ethanol sclerotherapy prior to IVF (Clinical Trials Number: NCT05962775) will be crucial in determining its role in this patient population. Future research should focus on optimizing the ethanol sclerotherapy protocol to balance efficacy and safety, and on exploring long-term outcomes in a larger cohort of infertile patients.

## Figures and Tables

**Table 1:** Studies evaluating the IVF cycle outcome after ethanol sclerotherapy compared with cystectomy and/or endometrioma in situ.

First Author, Year	Country	Design	Technique/Duration of Ethanol Exposure	Size (mm)	Cystectomy (N)	EST (N)	In Situ (N)	Outcome
Cystectomy vs. EST								
Yazbeck, 2009 [30]	France	Prospective	TV/10 min	20–60	26	35	-	CPR 19.2% vs. **48.3%**
Alborzi, 2021 [32]	Iran	Prospective	TV/Retention	30–60	57	44	-	LBR 38.6% vs. **29.5%**
Antonaci, 2022 [33]	Italy	Retrospective	TV/10 min	30–100	26	23	-	LBR 14.3% vs. **36.5%**
EST vs. Endometrioma in situ								
Aflatoonian, 2013 [34]	Iran	RCT	TV/10 min	>30	-	20	20	CPR **27.8%** vs. 15%
Miquel, 2020 [35]	Italy	Retrospective	TV/10 min	25–65	-	37	37	LBR **31.3%** vs. 14.5%
Rabattu, 2024 [36]	France	Retrospective	TV/10 min	25–80	-	34	33	LBR **34.8%** vs. 18%
Cystectomy vs. EST vs. Endometrioma in situ								
Lee, 2014 [31]	Korea	Retrospective	TV/Washing	>30	36	29	36	LBR 33.3% vs. **40.7%** vs. 33.3%

Note: The outcome rates that belong to EST are presented in bold. EST: ethanol sclerotheraphy; RCT: randomized controlled trial; TV: transvaginal ultrasound guided; CPR: clinical pregnancy rate; LBR: live birth rate.

**Table 2:** Studies reporting the spontaneous pregnancy rates after ethanol sclerotherapy.

First Author, Year	Country	Design	Technique/Duration of Ethanol Exposure	Size (mm)	Infertility or Fertility Desire (N)	Spontaneous PR
Noma, 2001 [43]	Japan	Retrospective	TV/case by case	NA	23	52.1%
Ikuta, 2006 [44]	Japan	Retrospective	TV/5 min	NA	9	44.4%
De Cicco Nardone, 2020 [45]	Italy	Retrospective	LS/15 min	40–100	28	50%
Martinez-Garcia, 2021 [46]	Spain	Retrospective	TV-TA/15 min	35–100	7	42.8%
Azizova, 2024 [42]	Türkiye	Retrospective	TV-TA/15 min	>40	11	45%

Note: PR: pregnancy rate; TV: transvaginal ultrasound guided; TA: transabdominal ultrasound guided; NA: not available.

**Table 3:** Studies evaluating the efficacy of ethanol sclerotherapy regarding serum AMH level change and/or recurrence rate.

First Author, Year	Country	Design	Technique/Duration of Ethanol Exposure	Size (mm)	EST (N)	AMH	Recurrence Rate	Compared to
Noma, 2001 [43]	Japan	Retrospective	TV/case by case	NA	74	NA	14.9%	Cystectomy Recurrence: 3.8%
Ikuta, 2001 [44]	Japan	Retrospective	TV/5 min	NA	18	NA	11.1%	-
Koike, 2002 [54]	Japan	Retrospective	TV/5 min	37–68	45	NA	13.3%	-
Hsieh, 2009 [55]	Taiwan	Retrospective	TV/Washing-10 min-Retention	>30	108	NA	26.9%	-
Yazbeck, 2009 [30]	France	Retrospective	TV/10 min	20–60	35	NA	12.9%	-
Gatta, 2010 [56]	Italy	Retrospective	TA-TV/10 min	20–50	54	NA	8%	-
Aflatoonian, 2013 [34]	Iran	RCT	TV/10 min	>30	20	NA	20%	Endometrioma in situ Similar AFC
Lee, 2014 [31]	Korea	Retrospective	TV/Washing	>30	29	NA	-	Cystectomy Lower AFC
Garcia-Tejedor, 2015 [57]	Spain	Prospective	TA-TV/15 min	40–100	27	NA	12%	-
Han, 2018 [58]	Korea	Prospective	TV/20 min	>30	14	No significant change	0%	-
Garcia-Tejedor, 2020 [50]	Spain	Prospective	TV/15 min	23–100	17	NS	5.9%	Cystectomy Recurrence: 28.6%
De Cicco Nardone, 2020 [45]	Italy	Retrospective	LS/15 min	40–100	53	NA	9%	-
Aflatoonian, 2020 [59]	Iran	Retrospective	TV/10 min–Retention	NA	43	NA	50%	-
Koo, 2021 [51]	Korea	Retrospective	TV/20 min	30–100	20	No significant change	0%	Cystectomy Recurrence: 7.8% AMH: Significantly decreased
Alborzi, 2021 [32]	Iran	Prospective	TV/Retention	30–60	44	No significant change	34.1%	Cystectomy Recurrence: 14%
Lee, 2022 [60]	Korea	Retrospective	TV-TA/20 min	>30	18	No significant change	5.5%	-
Antonaci, 2022 [33]	Italy	Retrospective	TV/10 min	30–100	23	No significant change	0%	Cystectomy Recurrence: 11.5% AMH: Significantly decreased
Ghasemi Tehrani, 2022 [52]	Iran	RCT	TV/20 min	40–100	35	No significant change	22.8%	Cystectomy Recurrence: 28.8% AMH: Significantly decreased
Crestani, 2023 [61]	France	Retrospective	LS/10 min	>40	69	Decreased slightly	11.8%	-
Vaduva, 2023 [62]	Romania	Retrospective	TV/7 min	30–80	54	No significant change	7.4%	Cystectomy AMH: Significantly decreased
Mohtashami, 2024 [53]	Iran	Retrospective	TV/10 min	>30	44	No significant change	11.4%	Cystectomy Recurrence: 15.9% AMH: Significantly decreased
Zeng, 2024 [63]	Korea	Prospective	TV-TA/15 min	>40	22	No significant change	0%	-
Azizoa, 2024 [42]	Türkiye	Prospective	TV-TA/15 min	>40	68	No significant change	2.9%	-
Rabattu, 2024 [36]	France	Retrospective	TV/10 min	25–80	34	NA	20%	-

Note: AMH: anti-Müllerian hormone; AFC: antral follicle count; RCT: randomized controlled trial; TV: transvaginal ultrasound guided; TA: transabdominal ultrasound guided; LS: laparoscopic; NA: not available.

## Data Availability

No new data were created or analyzed in this study. Data sharing is not applicable to this article.
